# Physical activity, resilience, and mental health in adolescents: a systematic review of recent evidence

**DOI:** 10.3389/fspor.2026.1757784

**Published:** 2026-06-03

**Authors:** Li Zhuang, Qiuju Wang, Yan Liu, Yijie Shi, Rujun Lv, Junping Liang, Ying Zhou, Mariusz Lipowski

**Affiliations:** 1 Guangdong Vocational College of Science and Technology, Zhuhai, China; 2 Jingyang Primary School, Zhuhai High-tech Zone, Zhuhai, China; 3 Zhuhai High-tech Zone Zhongshan University Affiliated Houhuan Primary School, Zhuhai, China; 4 WSB Merito University in Gdańsk, Gdańsk, Poland

**Keywords:** adolescents, anxiety, depression, mental health, physical activity, resilience

## Abstract

**Background:**

Adolescent mental health remains a global priority. Recent studies suggest that physical activity (PA) benefits mental health in youth, with psychological resilience increasingly recognized as a key explanatory pathway.

**Objective:**

This systematic review synthesizes recent evidence published between January 2022 and December 2025 on the relationships among physical activity, resilience, and adolescent mental health.

**Participants:**

Adolescents (approximately ages 10–19) from observational cohorts and intervention trials in community and at-risk samples included in the reviewed literature.

**Methods:**

Following PRISMA guidance, we systematically searched PubMed, Scopus, Web of Science, PsycINFO, and SPORTDiscus (January 2022–December 2025) using terms for PA, resilience, and adolescent mental health; reference lists were hand-searched. Eligible studies were quantitative and examined PA with resilience and mental health outcomes in adolescents. We conducted a best-evidence narrative synthesis given heterogeneity in designs and measures.

**Results:**

Across the reviewed evidence, higher physical activity levels were generally associated with better mental health outcomes and greater resilience in adolescents. A total of 10 studies formally tested mediation involving resilience or related psychosocial pathways, and all reported significant indirect effects.

**Conclusions:**

Current evidence supports PA as a scalable strategy to promote adolescent mental health, with resilience as a central mechanism. Programs that emphasize mastery, social connection, and supportive climates are likely to maximize gains. Future work should prioritize longitudinal/causal designs, harmonized measurement of resilience and outcomes, and equity-focused implementation to determine for whom and under what conditions PA most effectively fosters mental well-being.

## 1 Introduction

Adolescence is a pivotal developmental stage characterized by both opportunity and vulnerability (1, 2). During this time, mental health problems often emerge: globally, around 14% of 10–19-year-olds experience a mental health condition, with depression and anxiety among the leading causes of illness in youth (3, 4). By 2030, depression is projected to be the top cause of youth disability (3, 4). Concerningly, over 80% of adolescents worldwide do not meet recommended physical activity levels, and physical activity tends to decline during adolescence (1, 2, 5–7). Low activity may compound mental health risks in this sensitive period, given exercise's potential benefits for brain and psychological development (1, 2, 5).

Substantial evidence links regular physical activity with better adolescent mental health outcomes (8–11). Large-scale reviews and meta-analyses involving tens of thousands of participants have found that youth who are more physically active exhibit lower depression and anxiety symptoms and fewer externalizing problems (such as conduct issues or substance use) (10, 11). For example, a recent umbrella review reported that exercise interventions produce moderate reductions in depressive and anxiety symptoms in young people compared to inactive controls (12, 13). These benefits appear across diverse adolescent populations (including both clinical and general samples) (12–14), and certain activity modalities may be especially effective (e.g., combined aerobic–strength programs for depression, or resistance training for anxiety) (13, 15). Overall, physical activity is increasingly recognized as a promising non-pharmacological strategy to improve adolescents' emotional well-being and reduce the risk of psychopathology (16–18).

Psychological resilience has emerged as a key factor linking physical activity to mental health in adolescence. Resilience is broadly defined as the capacity to withstand and adapt to stressors or adversity (1, 2, 13, 19, 20). Higher resilience is associated with better coping skills, emotional regulation, and reduced risk of mental illness in youth (8, 21, 22). Importantly, resilience is not a fixed trait; it can be strengthened over time through positive experiences and behaviors (22–24). Physical activity, as a challenging but rewarding behavior, is hypothesized to build resilience by developing self-regulation skills and stress tolerance (25–27). Indeed, more physically active adolescents consistently report greater resilience or “mental toughness” and better coping ability, which correlates with better mental health adjustment (22, 28, 29).

Physical activity likely promotes adolescent mental health through multiple interconnected pathways—both psychosocial and biological—with resilience as a central hub (11, 30, 31). Psychosocially, exercise provides experiences of mastery and social connection that enhance self-efficacy, self-esteem, and support from peers (11, 32, 33). These psychosocial resources facilitate stress management and emotion regulation, contributing to lower depression and anxiety (11, 34, 35). Biologically, regular moderate-intensity exercise induces neurochemical changes (e.g., endorphin release, increased BDNF), regulates HPA-axis stress responses and inflammation, and improves sleep quality (4, 36, 37). During adolescence—a period of heightened neural plasticity—these changes support brain regions (such as the prefrontal cortex) involved in cognitive control of emotion (25, 27). Thus, physical activity can improve mental health both directly (via immediate mood and physiological benefits) and indirectly (by strengthening resilience and related psychosocial mechanisms).

Given the public health importance of adolescent mental well-being and the modifiable nature of physical activity, a timely systematic review of the triadic relationship between physical activity, resilience, and mental health is needed. Previous reviews have documented the mental health benefits of youth physical activity and suggested candidate mechanisms, but questions remain about how and for whom these benefits occur (11, 38, 39). In particular, a surge of research in the past 3–5 years—including large longitudinal studies and new systematic reviews—warrants an updated synthesis focusing on resilience as a pathway. Accordingly, this review aims to: (1) evaluate recent evidence (2022–2025) linking adolescent physical activity participation to mental health outcomes; (2) examine the role of psychological resilience as both an outcome and a mediator in this relationship; and (3) explore moderators and subgroup differences (e.g., gender, culture, risk level) that may influence the physical activity–mental health link. By integrating findings from high-quality recent studies, we seek to clarify how physical activity contributes to adolescent mental well-being, determine which subgroups and contexts show the strongest effects, and highlight implications for theory and intervention.

## 2 Methods

This review was conducted in accordance with PRISMA (Preferred Reporting Items for Systematic Reviews and Meta-Analyses) guidelines (22, 40). We systematically searched PubMed, Scopus, Web of Science, PsycINFO, and SPORTDiscus for articles published from January 2022 through December 2025. The search strategy combined keywords related to physical activity (e.g., “physical activity,” “exercise,” “sport”), resilience (e.g., “resilience,” “mental toughness,” “coping”), and adolescent mental health (e.g., “depression,” “anxiety,” “well-being,” “adolescent”). We also hand-searched reference lists of relevant papers to ensure comprehensive coverage.

Studies were eligible if they were quantitative empirical investigations (observational or intervention) examining associations between physical activity, psychological resilience (or related constructs), and mental health outcomes in adolescents. We included general population samples and studies of clinical youth provided mental health or resilience outcomes were reported. Non-empirical papers, qualitative studies, and case reports were excluded. We also excluded studies focusing on adolescents with serious medical conditions (e.g., diabetes) unless the study's focus was on mental health in otherwise healthy youth. After removing duplicates, two reviewers independently screened all titles and abstracts; full-texts of potentially eligible articles were then evaluated against the inclusion criteria, with disagreements resolved by a third reviewer. This review was designed as a systematic review of recent primary empirical studies rather than an umbrella review. Recent systematic reviews and meta-analyses were consulted to contextualize the broader evidence base, but they were analyzed separately from the primary empirical studies in order to avoid duplicate counting of evidence. Accordingly, primary empirical studies, secondary review-level evidence, and background sources were treated as distinct evidence categories throughout the synthesis.

**Figure 1:**
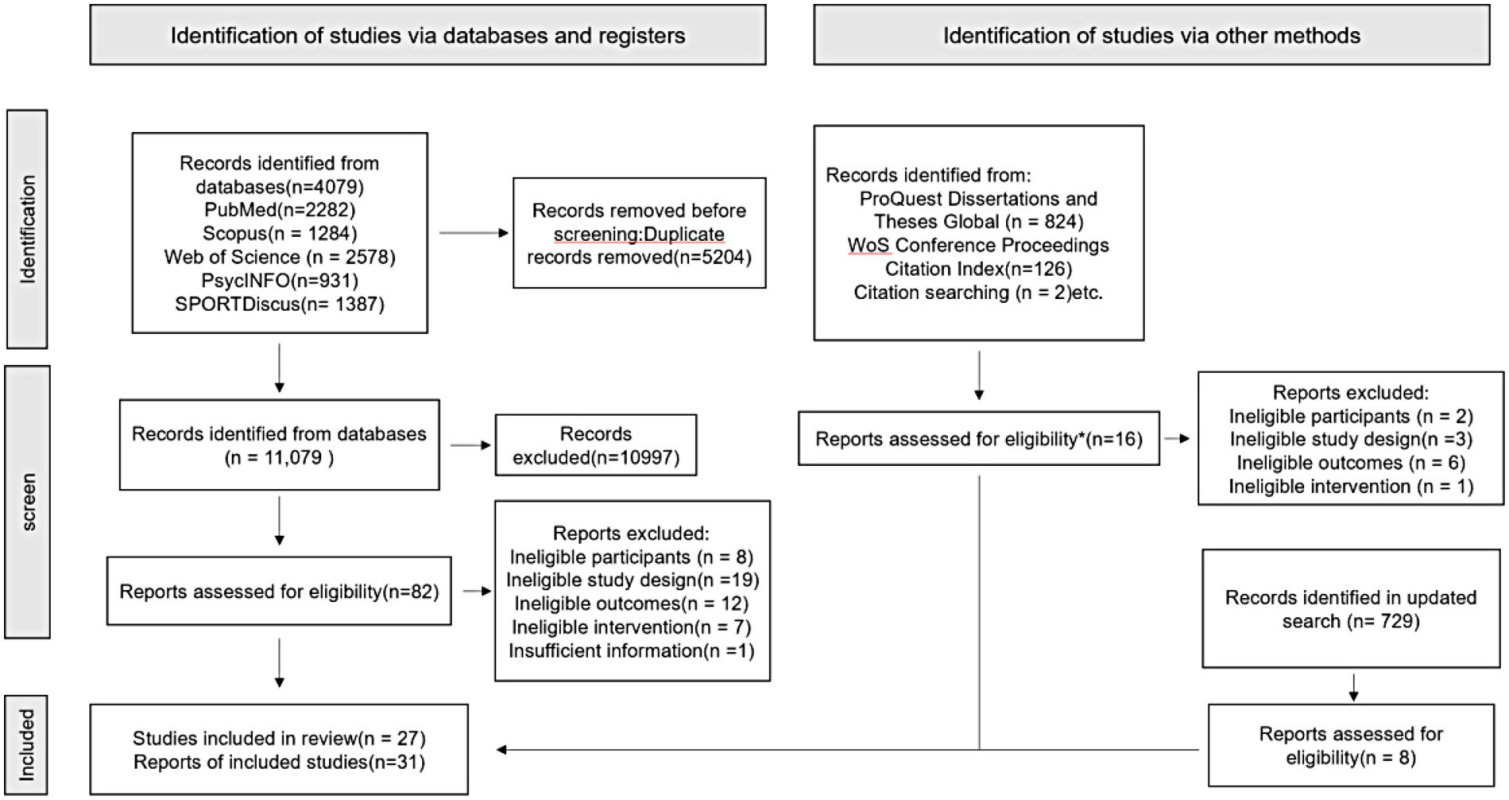
PRISMA flow diagram.

Our searches yielded approximately 11,000 records. primary empirical studies, secondary review-level evidence, and contextual/background sources are reported separately to ensure internal consistency across the PRISMA flow diagram (Figure 1), study classification, and evidence tables. For each included study, we extracted key details: sample characteristics (age range, sex, location, any special population), study design, measures of physical activity (e.g., self-reported moderate-to-vigorous activity minutes, sport participation frequency, or fitness test performance such as VO₂max) (41–43), measures of resilience (standardized resilience scales or proxies like “mental toughness”), mental health outcomes (e.g., depressive symptoms, anxiety, stress, well-being), and main findings related to the physical activity–resilience–mental health relationship (22, 44, 45). For intervention studies, we additionally noted the type of physical activity (aerobic, resistance, team-based, mind-body, etc.), frequency/duration, and changes in mental health or resilience from pre- to post-intervention.

Study quality was appraised using the QualSyst tool for quantitative studies (22, 46), assessing aspects such as study design, sample bias, outcome measurement validity, and analytical rigor. Each study received a quality score (percentage of criteria met) and was rated as excellent, good, fair, or poor (22, 46). Overall, most studies were of moderate-to-high quality; common limitations included reliance on cross-sectional designs, self-reported physical activity (prone to bias), and inconsistent control of confounders like baseline mental health or socioeconomic status. When synthesizing the evidence, we placed greater weight on findings from longitudinal and experimental studies and on results from systematic reviews with rigorous methods, while treating exploratory or lower-quality findings with caution. No formal meta-analysis was performed due to heterogeneity in measures and outcomes. Instead, we conducted a narrative, best-evidence synthesis (11, 47, 48), grouping findings by key themes (e.g., mental health outcomes, resilience outcomes, mediators, moderators) and evaluating the consistency of results across studies.

## 3 Results

### 3.1 Overview of included studies

Across the reviewed evidence base, higher physical activity levels were generally associated with better mental health outcomes in adolescents, while secondary review-level evidence supported similar broader patterns. (Table 1 in the Supplementary material summarizes key characteristics of representative studies.) Collectively, the reviewed studies encompassed data from over 500,000 adolescents worldwide. Mental health outcomes assessed ranged from internalizing symptoms (depression, anxiety, stress, general psychological distress) to externalizing behaviors (conduct problems, hyperactivity), as well as positive indicators like well-being and life satisfaction. Physical activity was measured in various ways, including self-reported moderate-to-vigorous physical activity (MVPA) minutes per day, frequency of sports participation, fitness test performance (e.g., VO₂max for aerobic fitness) (41–43), or participation in specific exercise programs. Psychological resilience was assessed in a subset of studies using standardized scales (such as the Connor–Davidson Resilience Scale) or via related constructs (e.g., “mental toughness” or coping efficacy).

**Table 1:** Representative evidence sources cited in the synthesis, classified by evidence type.

No	Author/Year	Evidence type	Study design/source type	Sample/scope	Main relevance to the review	Included in primary synthesis?
1	Singh et al. (13)	Secondary review/meta-analysis	Umbrella review and meta-meta-analysis	Pooled evidence across multiple meta-analyses of children and adolescents	Shows that exercise significantly reduces depressive and anxiety symptoms; supports PA as part of comprehensive mental health care for youth	No—secondary evidence only
2	White et al. (11)	Secondary review/meta-analysis	Systematic review and synthesis of best evidence (study length varies by included study)	Multi-study review	Self-esteem, self-efficacy, emotional and social support are key mediators; gender and social support may moderate effects.	No—secondary evidence only
3	Qiu et al. (22)	Secondary review/meta-analysis	Systematic review and meta-analysis (*k* ≈ 21)	Aggregated data from about 21 studies of young students	Physical activity is positively associated with psychological resilience, with a pooled standardized coefficient of *β* ≈ 0.27.	No—secondary evidence only
4	Zhang et al. (4)	Primary empirical study	Cross-sectional online survey; single time-point assessment	*N* = 400 (Chinese middle school students; mean age = 13.74 years)	Physical activity, self-efficacy, stress management, and mental health are significantly correlated; self-efficacy and stress management mediate these effects.	Yes
5	da Silva et al. (49)	Primary empirical study	Cluster randomized controlled trial; 12-week duration	*N* = 306 (Brazilian secondary school students; intervention *n* = 165, control *n* = 141)	No overall group differences, but significant reductions in depression and anxiety were observed in adolescents with moderate-to-severe baseline symptoms; physical activity supports maintenance of psychological well-being.	Yes
6	Shi et al. (50)	Primary empirical study	Observational/interventional analyses (synthesis of published reports; duration varied across studies)	Adolescent sample (details reported in original study)	Included because it addresses the PA–resilience–mental health pathway;	Yes
7	Duncan et al. (51)	Primary empirical study	Cross-sectional/longitudinal analyses (dataset-dependent)	Adolescent sample (details reported in original study)	Meeting the 24-hour movement behavior guidelines is associated with greater well-being and lower depression/anxiety.	Yes
8	Biadgilign et al. (91)	Primary empirical study or miscoded entry	Cross-sectional, population-representative sample	*N* = 632 (child–adolescent and parent dyads)	Current row appears mismatched: the title repeats the Jiang et al. university-student paper, but the sample describes child–adolescent dyads; this entry must be corrected before retention	Yes
9	World Health Organization (7)	Background/contextual source	Authoritative compendium (non-empirical; not a primary study)	Not a primary empirical study	Provides contextual global data showing that more than 80% of adolescents do not meet recommended activity levels	No—contextual only
10	Patton et al. (1)	Secondary review/background review	Narrative/systematic review	Broad review of adolescent PA behaviors	Used to contextualize the widespread prevalence and implications of insufficient PA in adolescence	No—contextual/secondary only
11	World Economic Forum (85)	Background/contextual source	News/interpretive commentary (non-research)	Not a primary empirical study	Provides broad contextual commentary about adolescent inactivity and health risk	No—contextual only

Approximately two-thirds of the empirical studies reported a significant positive association between physical activity and adolescent mental health indicators (i.e., more active youth had fewer mental health problems and/or higher well-being) after controlling for confounding factors (22, 28, 52). The strength of association was typically small-to-moderate. For instance, a 2025 meta-analysis of 21 studies found an average correlation of about r ≈ 0.25 between physical activity and resilience in young people (22, 28, 53), and resilience in turn was positively correlated with mental well-being in those studies. Longitudinal studies similarly showed that adolescents who maintained higher physical activity over time tended to report lower subsequent depressive symptoms and stress compared to less active peers (54–56). Notably, in one longitudinal cohort of ∼300 adolescents assessed during the COVID-19 pandemic, baseline physical activity predicted lower psychological distress 6 months later, and this effect was fully mediated by higher baseline self-esteem (54–56). This finding suggests that the mental health benefits of physical activity may be partly explained by improvements in psychosocial resources such as self-esteem (a component of resilience).

Intervention studies further support the beneficial impact of activity. Around 15 trials in our review examined structured physical activity programs for adolescents and generally found significant reductions in depression and anxiety symptoms, along with improved positive well-being—especially among youths with elevated baseline symptoms. For example, a 12-week school-based exercise program in the UK led to clinically meaningful decreases in anxiety and depression scores in adolescents who initially had moderate-to-severe symptoms (11, 49). Similarly, a randomized controlled trial in China found that adding a structured aerobic exercise routine improved adolescents' self-reported resilience and reduced perceived stress compared to a no-exercise control group (50, 57). Although most trials focused on symptom reduction, a few also measured resilience as an outcome and observed increases in resilience scores following the physical activity intervention (50, 57). However, not all interventions were uniformly effective; results sometimes depended on program characteristics. For instance, interventions emphasizing team sports and cooperative physical challenges often yielded gains in social connectedness and resilience, whereas purely individual exercise regimens sometimes produced smaller or no psychosocial benefits—especially for youths who lacked social support for exercise. Table 1 presents representative evidence sources cited in the synthesis and classifies them by evidence type. It is not intended to provide a complete list of all included studies. Primary empirical studies, secondary review-level evidence, and background sources are shown together here for transparency.

### 3.2 Physical activity and mental health outcomes

For internalizing problems, numerous studies found that more physically active adolescents report fewer depressive and anxiety symptoms and lower odds of developing mood or anxiety disorders (13, 14, 58). Meta-analytic evidence indicates a moderate beneficial effect of exercise on youth anxiety (standardized mean difference ≈ −0.39) (13, 14, 58). Certain activity types may be particularly helpful for anxiety reduction: for example, resistance exercise and mind-body practices like yoga have shown promise, possibly by combining physical exertion with relaxation or confidence-building elements (13, 59). Regular physical activity is also associated with lower perceived stress and better mood regulation in adolescents. Several studies observed that higher PA was linked to lower stress levels and better stress-coping skills (4, 60, 61). In one cross-sectional study of ∼400 middle-schoolers, the entire association between physical activity and a composite mental health score (low distress, high life satisfaction) was mediated by higher self-efficacy and effective stress-coping behaviors in the active group (4, 61, 62). This underscores that physically active youth may handle stressors more effectively, thereby maintaining better overall mental health.

Physical activity appears beneficial for externalizing and behavioral outcomes as well, though this area is less extensively studied. Some evidence suggests that active adolescents exhibit fewer conduct problems and less hyperactivity/inattention (10, 11, 17, 18). In particular, youths with attention-deficit/hyperactivity disorder (ADHD) may experience improved concentration and reduced impulsivity with regular exercise, likely through enhancements in executive function and self-regulation. A recent review identified exercise as a promising adjunct treatment for externalizing disorders, including ADHD (16–18). Additionally, one longitudinal study reported that adolescents participating in consistent sports activities had lower odds of substance use and other risky behaviors over time, suggesting that involvement in positive physical outlets can have a protective effect. However, the evidence for externalizing outcomes is still emerging and somewhat mixed—some studies found no strong link after accounting for confounders (8, 9). More targeted research is needed on how physical activity influences behavioral and cognitive aspects of mental health in teens.

Not all forms of physical activity confer equal mental health benefits; the context of activity is pivotal. For example, one study found that adolescents engaging exclusively in solitary exercise (exercising alone) actually reported higher depression and anxiety levels than those who did not exercise at all (4, 63, 64). Similarly, youth prone to rumination experienced heightened negative emotion during solitary workouts in an experimental setting (4, 63, 64). In contrast, activities that involve social interaction or team participation often provide social support and enjoyment that amplify mental health benefits (11, 34, 35). These findings suggest that simply being active is not a panacea—*how* and *with whom* adolescents are active matters. A supportive, enjoyable exercise environment (e.g., playing on a sports team with friends or engaging in group exercise led by an encouraging instructor) can foster a sense of belonging and accomplishment, thereby enhancing the positive impact on mood and mental health. Conversely, exercise undertaken in isolation or under pressure may yield fewer benefits and could even be counterproductive for some vulnerable youth (4, 63, 64). This theme of context and social environment moderating outcomes is revisited in the discussion of subgroup differences.

### 3.3 Physical activity and psychological resilience

A central finding of this review is the robust positive link between adolescents' physical activity and their psychological resilience. In the first meta-analysis focused on this topic, Zhao et al. (65) synthesized 21 studies and found that more physically active youth scored significantly higher on resilience measures (pooled standardized effect size ∼0.20–0.30) than less active youth (22, 53). This association was evident in both adolescent and young adult samples, though it was stronger in adolescents (∼0.30) than in college-aged youth (∼0.23) (22), suggesting that early-to-mid adolescence may be a particularly sensitive period during which activity behaviors influence resilience development (22, 65). Notably, all 21 studies in that meta-analysis reported a positive correlation between physical activity level and resilience (none found a negative relationship) (22, 28, 66), underscoring remarkably consistent evidence across diverse contexts.

Why might exercise and resilience go hand-in-hand? Engaging in physical activities—especially those that are moderately challenging—provides opportunities for youth to develop coping skills, perseverance, and confidence under pressure (21, 22). For example, adolescents who regularly play sports or exercise often learn to overcome obstacles, handle setbacks, and achieve goals, which can translate to greater resilience in daily life (22, 28, 53). Empirical studies support this: physically active adolescents tend to report better stress-coping ability and a more optimistic, determined mindset compared to their sedentary peers (8, 22). One study of high school students found that those meeting the recommended PA level (≥60 min/day) scored significantly higher on resilience scales (measuring problem-solving, emotional regulation, etc.) than those who were inactive (22, 28, 53). Longitudinal data also hint that maintaining fitness leads to gains in resilience over time. Intervention research suggests that deliberately incorporating physical challenges and teamwork (e.g., outdoor adventure programs, sports camps) can improve resilience in at-risk adolescents. Although relatively few RCTs have measured resilience as an outcome, some have reported moderate resilience improvements after multi-week physical activity programs emphasizing personal development and coping skills. These findings align with qualitative reports from teens that exercise makes them feel more confident in their ability to handle difficulties, reflecting an enhanced sense of resilience.

Not only is resilience improved by physical activity, it also serves as a mediator in the pathway from activity to mental health. A recent systematic review of mediation studies found strong evidence that resilience mediates the effect of physical activity on various mental health outcomes (11, 53). Recent review-level evidence indicates that 10 studies formally tested resilience as a mediator, and all reported significant indirect effects linking physical activity to better mental health outcomes. (11, 53). In practical terms, this means part of the reason physically active adolescents have fewer depressive or anxious symptoms is that exercise helps them develop higher resilience (better coping skills, optimism, perseverance), which in turn directly contributes to lower psychological distress (11, 53, 67). For instance, in a study of children with ADHD, regular moderate-to-vigorous exercise led to improved psychological well-being through increased resilience—children who exercised more became more resilient, and that boost in resilience was associated with reductions in emotional problems (11, 68, 69). Similarly, a study of Hong Kong adolescents found that resilience significantly mediated the positive effects of physical activity on mental health and life satisfaction (11, 63, 70). These results establish resilience as a key mechanistic link: by bolstering resilience, physical activity confers protection against mental health problems.

Even so, there are nuances to explore regarding *how* different types of physical activity optimally build resilience. Sports psychology observations suggest that organized sports can foster resilience via exposure to competition and teamwork (11, 64). Beyond sports, however, evidence is limited on which exercise contexts or approaches best cultivate resilience (11, 64, 71). Some researchers hypothesize that mastery experiences are crucial—when youths learn new physical skills or improve their fitness, they gain a sense of competence that carries over to general resilience (11, 32, 61). Training environments emphasizing personal improvement, achievable challenges, and supportive coaching (while avoiding excessive pressure) are likely ideal for resilience-building (11, 61, 66). Social factors also play a role: supportive coaches and positive team climates provide a safe environment to struggle and recover, reinforcing adaptive coping. Overall, our review confirms that active adolescents are more resilient, and that resilience is a key pathway by which physical activity benefits mental health. Future research should continue examining which exercise modalities and contexts most effectively promote resilience in youth (see Future Directions).

#### 3.3.1 Mechanisms linking physical activity, resilience, and mental health

Notably, several studies identified mediating pathways that help explain why physical activity benefits adolescent mental health. In particular, improvements in psychosocial factors such as self-esteem, self-efficacy, and social support were frequently found to mediate the relationship between exercise and reduced depression/anxiety, indicating that physical activity boosts adolescents' confidence and social connectedness, which in turn improves mental well-being. Biological mechanisms were also implicated: regular exercise is associated with healthier stress physiology (e.g., more adaptive cortisol responses) and neurobiological changes (like increased neurotrophic factors), which can make the brain more resilient to stress. These converging pathways support the view that physical activity enhances mental health both directly and indirectly, by building a more resilient youth both psychologically and physiologically.

#### 3.3.2 Moderators and subgroup differences

Adolescents are not a homogeneous group—the effects of physical activity on mental health and resilience can vary across subpopulations. Key moderators identified in the literature include:

- Baseline Mental Health Status: Adolescents with higher baseline mental health symptoms tend to derive greater mental health benefits from physical activity than those who start out mentally healthy (11). This implies that at-risk youth (who need it most) often see the biggest improvements—a promising implication for targeting interventions.
- Gender: Some studies suggest the PA–mental health link is stronger in girls (perhaps because increasing their activity addresses a larger deficit in well-being) (72, 73), whereas others find no significant gender difference once overall activity levels and other factors are accounted for (11). Overall, evidence is mixed (11). Both boys and girls benefit from being active, but since girls are often less active and more prone to depression/anxiety, special efforts to engage them in exercise are crucial to improve their mental health outcomes (74).
- Socioeconomic Status (SES): Socioeconomic factors might moderate outcomes, but evidence is scarce and inconclusive (11). Some data suggest lower-SES adolescents may gain relatively larger mental health benefits from structured exercise programs (because they start with higher stress and have fewer resources), but low-SES youth also face more barriers to exercise (cost, safety, lack of facilities). Ensuring equitable access to physical activity (e.g., free or school-based programs in low-income areas) could especially help disadvantaged youth. More research focusing on low-SES groups is needed.
- Cultural Context: Cultural norms shape physical activity patterns and their mental health effects. In cultures where exercise is often solitary (done out of duty or academic pressure), it may confer fewer mental health benefits, whereas in cultures that promote social, enjoyable exercise (e.g., team sports), the benefits tend to be greater (4, 63, 75). Tailoring physical activity programs to fit local cultural preferences (e.g., offering dance in cultures that value dance, martial arts where appropriate) can improve engagement and outcomes.
- Individual Differences: Factors like fitness level, personality, and genetics may influence who benefits most from exercise. For example, one study found adolescents with higher baseline fitness and resilience showed greater mood improvements with exercise, whereas very unfit youth may need a ramp-up period to see benefits (11, 76, 77). Personality might also play a role (an extravert might gain more from the social aspect of team sports, while a highly anxious teen might initially find exercise stressful but benefit over time). Preliminary research even hints that genetic differences (e.g., in dopamine or serotonin pathways) could moderate psychological responses to exercise (11, 78, 79), though this is not well studied in adolescents.

In summary, while physical activity and resilience generally benefit adolescents across the board, context matters. Those with greater initial needs (like at-risk youth) often benefit most, making them priority targets for interventions (11). Meanwhile, increasing engagement among low-activity groups such as girls and disadvantaged youth is essential to ensure they do not miss out on the mental health benefits of exercise (74). Overall, socially supportive and culturally appropriate activities tend to maximize positive outcomes for all, underscoring that *how* and *with whom* adolescents are active can shape the mental health impact (4, 63, 64).

## 4 Discussion

This systematic review set out to clarify the relationships between physical activity, psychological resilience, and adolescent mental health by synthesizing the latest evidence (2022–2025). The findings provide compelling support for physical activity as a positive influence on adolescent mental health globally, and they highlight resilience as a key mechanism in this link. Our conclusions reinforce and extend prior research, showing that the association between physical activity and mental well-being holds true in recent large samples and trials, and that it is at least partly mediated by improvements in resilience (and related psychosocial factors). These results align with earlier reviews and meta-analyses that have long suggested exercise's mood-enhancing and stress-reducing effects in young people (10, 11, 13, 14, 17, 18, 80). Notably, during the COVID-19 pandemic, adolescents who stayed physically active showed smaller increases in anxiety and depression and coped better with lockdowns compared to less active peers (56, 81, 82). This real-world example underscores the protective value of physical activity in times of adversity, likely because it provided youth with outlets for stress, structure, and social connection when those were otherwise disrupted. In short, promoting physical activity should be considered a viable strategy to help address the ongoing youth mental health crisis exacerbated by global stressors.

A major contribution of this review is elucidating *how* physical activity translates into mental health benefits, with resilience emerging as the linchpin. The evidence supports a multicomponent resilience-building pathway: being active helps cultivate a more resilient mindset and physiology in adolescents, and that resilience buffers against psychological distress (11, 53). This dovetails with theoretical frameworks such as the “Resilience Model of Physical Activity,” which proposes that exercise acts as a manageable stressor that trains the body and mind to handle other stressors more effectively (similar to building muscle by gradually lifting heavier weights) (4, 22, 23). Our synthesis suggests that the “training” provided by exercise is indeed multifaceted—ranging from boosting self-esteem (physical challenges mastered build confidence) to improving neurobiological stress regulation (regular exercise may moderate cortisol responses). It's noteworthy that resilience in the included studies was often defined broadly, overlapping with constructs like self-efficacy, coping ability, or social adaptability. Physical activity touches on so many of these facets—physical mastery, cognitive focus, emotional regulation, social bonding—that it is uniquely positioned to enhance overall resilience. Few interventions in mental health can simultaneously target all these dimensions; exercise is one of the rare interventions that can.

Our findings also imply a positive feedback loop between physical activity and mental health through resilience. If being active builds resilience and better mental health, adolescents who enjoy improved mental well-being (in part due to higher resilience) may be more inclined to remain active, creating a virtuous cycle. Conversely, poor mental health can lead to inactivity (due to low energy, motivation, or social withdrawal), which may further erode resilience—a vicious cycle for some youth (1, 2, 5). Longitudinal evidence supports this bidirectionality: for example, higher baseline depression predicts lower physical activity at follow-up, while higher baseline activity predicts lower depression later. Breaking this cycle is a key challenge and opportunity. Interventions could target either side—for instance, using physical activity programs to boost mood and resilience in struggling teens (thereby increasing the likelihood they remain active), and conversely providing mental health support to inactive or depressed adolescents to enable them to engage in exercise. Recognizing this two-way relationship is important for designing effective prevention and treatment strategies.

Our review underscores that context and individual differences moderate the benefits of physical activity, suggesting the need for personalized approaches. The strong influence of social support (with greater benefits from exercise when support is high) (11, 35, 83) indicates that physical activity should be framed as a social intervention as much as an individual behavior. This is consistent with social cognitive theory—an adolescent's environment (supportive relationships, role models) shapes how much they gain from healthy behaviors. Similarly, the finding that effects can be strongest in those with existing mental health issues (11) is encouraging, as it means physical activity can serve as an early intervention for youth showing signs of depression or anxiety. However, it also raises a practical caution: teens suffering from severe depression or anxiety may have difficulty initiating or sustaining an exercise routine, so they might require additional support (e.g., supervised programs integrated into mental health services) to fully benefit.

Notably, adolescent girls' physical activity levels are consistently lower than boys' (84, 85), which likely contributes to their higher rates of internalizing problems. Even if girls benefit from exercise as much as boys do, their under-participation means they currently miss out on important benefits; thus, finding ways to engage and retain girls in physical activity is crucial for reducing gender disparities in adolescent mental health.

Several limitations in the evidence base and in our review should be acknowledged. First, despite the inclusion of longitudinal data, many mediation analyses were cross-sectional, limiting causal inference. We often assume physical activity leads to increased resilience and better mental health, but it could also be that resilient, mentally healthy teens are more likely to be active. While the mediation models we reviewed are consistent with a pathway from physical activity to resilience to mental health, experimental studies manipulating resilience (or related mediators) would strengthen causal claims. Second, there was substantial heterogeneity in measurement of both exposures and outcomes across studies. “Physical activity” was quantified variously (e.g., step counts, self-rated activity frequency, fitness levels) and “mental health” ranged from diagnosed disorders to general well-being scales. This diversity shows the broad relevance of the topic but introduces noise and makes direct comparisons difficult. Future research should move toward more standardized measures or at least include multiple metrics (for example, self-report plus device-based activity tracking) to enhance comparability.

Publication bias is a concern, but an umbrella review with rigorous bias assessment still found positive effects of exercise on youth mental health, lending credibility to our conclusions (12, 13). To reduce the risk of duplicate counting, primary empirical studies were distinguished from secondary review-level evidence in the synthesis. Systematic reviews and meta-analyses were used only to contextualize overarching patterns and were not interpreted as equivalent to primary included studies in the main synthesis; however, we primarily used reviews to capture overarching patterns (e.g., mediator summaries) and relied on primary studies for specific data to minimize redundancy.

From a methodological standpoint, many exercise trials had small sample sizes or lacked blinded outcome assessment, which limits confidence in their results. Few recent trials accounted for important confounders like pubertal stage or objectively verified exercise adherence, even though puberty can influence both mental health and physical capacity. And while none of the reviewed studies reported serious adverse effects of exercise, future research should still monitor for potential negative outcomes (e.g., injury or unhealthy exercise behaviors) to ensure interventions remain safe.

Theoretical implications: Our findings resonate with multiple theoretical frameworks. For instance, behavioral activation theory (common in depression treatment) posits that increasing engagement in rewarding activities leads to improved mood; our review supports this principle, with the nuance that part of the “reward” of exercise is improved self-perception and resilience. Similarly, self-determination theory suggests that fulfilling basic needs for autonomy, competence, and relatedness through exercise enhances well-being, and indeed we observed that mastery experiences and social connection were key mediators of exercise benefits for mental health (11, 61, 66). This implies that exercise needs to foster an overall sense of capability in life, not just fitness-specific confidence, to maximally build resilience. Interventions should help adolescents transfer the confidence and skills gained via exercise to other life domains (for example, encouraging teens to apply the perseverance from sports to challenges in school or work).

From a neurodevelopmental perspective, the idea of exercise enhancing top-down cognitive control over emotion aligns with neuroplasticity models of adolescence. This period offers a window to shape neural circuits; consistent with our findings, exercise can be viewed as an enriching environmental input that strengthens neural networks (particularly in prefrontal regions) involved in self-regulation and stress management (25–27). This biobehavioral perspective invites interdisciplinary research—for example, combining neuroimaging with psychological assessments and fitness measures—to map precisely how moving the body benefits the developing brain and mind.

Practical applications: This review yields several actionable insights. Schools and youth programs should integrate physical activity into mental health promotion. Ensuring that adolescents have regular opportunities for enjoyable physical activity (through physical education, active recess, sports teams, clubs, etc.) can strengthen resilience and reduce common mental health problems. Likewise, healthcare providers can “prescribe” exercise as preventive medicine for at-risk youth or as an adjunct to therapy. For example, a physician might encourage a teen with mild depression to engage in a preferred activity multiple times per week and help connect them with local exercise resources. Given that benefits tend to be greatest in youth with existing symptoms (11), adding a structured exercise component to interventions for adolescents with depression or anxiety may enhance outcomes. It is crucial to frame such programs not just as exercise for fitness, but as *resilience training*—helping teens build confidence, coping skills, and social support through physical activity. Key mediators like self-efficacy, positive social connections, and mood improvement should be intentionally fostered (e.g., via group activities, goal-setting with feedback, and guided reflection on overcoming challenges).

Our review also highlights that exercise programs require intentional design to maximize resilience gains. A supportive atmosphere that emphasizes effort, improvement, and fun (rather than competition or comparison) is vital. Coaches and instructors should provide positive feedback and create opportunities for small “wins” that boost self-esteem. The importance of social support (11, 34, 35) means that adolescents should feel connected and encouraged—large, impersonal classes may be less effective than smaller groups or clubs where peers and mentors actively engage with participants. For youth who prefer solitary exercise, incorporating some social element (such as occasional group sessions or online communities where teens can share progress and receive encouragement) can ensure they still benefit from a sense of community.

While our narrative approach allowed us to integrate diverse findings, future quantitative syntheses and targeted studies are needed to build on this work (see Future Directions below). Meta-analyses could quantify the effect of exercise on resilience scores or the proportion of mental health benefits explained by resilience. More longitudinal mediation studies would clarify temporal ordering (e.g., measuring physical activity at Time 1, resilience at Time 2, and mental health at Time 3 to establish causal sequences). It will also be important to explore the optimal “dose” and type of activity for mental health benefits. Current evidence hints that moderate-intensity activity might yield the most mood benefit, and a combination of aerobic and resistance training could be ideal for depression, but the ideal frequency and duration remain unclear. Large-scale studies could investigate whether there is a threshold of activity {some suggest around 60 min of MVPA daily, consistent with guidelines (86)} beyond which additional mental health gains plateau. Moreover, qualitative research with adolescents can enrich our understanding of how they perceive the link between exercise and mental well-being, and why some may find exercise stressful (for instance, teens who ruminate might do better with guided group exercise to keep their mind positively engaged) (4, 63, 87). Addressing these nuances will be key to refining interventions.

## 5 Future directions

Building on the evidence map and gaps identified in this review, we offer eight concise, testable priorities to strengthen causal inference, clarify mechanisms, and improve real-world scalability in adolescent PA–resilience–mental health research.

- Longitudinal and Developmental Studies: Undertake multi-wave cohorts with causal modeling (e.g., cross-lagged, g-methods) to establish temporal ordering among physical activity, resilience, and mental health and to pinpoint sensitive periods from early to late adolescence (76, 88). Emerging findings suggest the PA–resilience–mental health link extends into young adulthood but may vary with maturation; stratified analyses can guide age-tailored intervention timing (22, 65).
- Mechanistic Research: Embed pre-registered mediation in RCTs to test whether improvements are accounted for by hypothesized pathways (e.g., self-esteem, social support), including multi-arm designs that add self-efficacy or social-support components to exercise (25, 26). Pair trials with feasible neuro/biological markers (e.g., prefrontal/hippocampal indices, cortisol, inflammatory markers) to validate psycho-bio-behavioral mechanisms (27).
- Diverse Activity Modalities and Contexts: Use factorial or SMART adaptive trials to compare modality (team vs. solo; aerobic vs. mind–body vs. martial arts), dose (frequency–intensity–duration), and context (green/outdoor, family-based, school-based). Estimate dose–response and incremental benefits (e.g., anxiety reduction with moderate intensity; added gains from nature or family involvement) to inform tailored prescriptions.
- Targeted Interventions for At-Risk Youth: Prioritize high-risk groups (early symptoms, high stress/ACEs, very low activity) with trials that test prevention and adjunctive treatment effects in school/community settings. Include low-SES and marginalized populations via culturally adapted, accessible delivery (on-site or digital), and conduct implementation evaluations that address barriers and sustainability.
- Gender and Inclusivity: Co-design programs that increase engagement and retention among girls and sexual/gender-minority youth (e.g., girls-only groups, music/dance elements, non-binary sport options). Evaluate safety, acceptability, and moderators of effect to ensure benefits extend to those least served by mainstream sport.
- Integrating Exercise into Mental Health Services: Test multicomponent interventions that embed structured exercise within school mental-health programs or therapy (e.g., CBT/resilience training + exercise), and compare integrated packages against single-component arms for symptom relief and relapse prevention. Align PE curricula with SEL objectives and track adherence and transfer.
- Technology and Innovation: Assess apps, wearables, and VR/exergames as adjuncts to bolster adherence, peer support, and skill rehearsal—while safeguarding data privacy and engaging caregivers. Report real-world usage trajectories and maintenance beyond initial novelty.
- Structural and Policy Approaches: Evaluate population-level changes—extended recess/PE, facility upgrades, subsidies—using natural experiments, interrupted time-series, difference-in-differences, or stepped-wedge cluster trials. Link WHO inactivity-reduction targets (15% by 2030) with youth mental-health indicators to guide large-scale implementation and scale-up (7, 89, 90).

## 6 Conclusion

Adolescence is a critical period for shaping lifelong mental health. Evidence synthesized in this review indicates that physical activity is an effective, evidence-based strategy for fostering resilience and preventing mental health problems in this population. Our review of recent studies found that adolescents who are more physically active consistently show better mental health outcomes (including lower levels of depression, anxiety, and stress, and higher well-being), with a substantial portion of these benefits mediated through enhanced resilience, self-efficacy, and social connectedness. Mechanistically, exercise acts as a “positive stressor” that strengthens adolescents' capacity to cope with challenges via dual pathways: *psychological* (boosting self-esteem, emotion regulation skills, and coping strategies) and *physiological* (promoting neuroplasticity and healthy stress physiology). In essence, regular physical activity helps build the inner strength—resilience—needed to navigate the stresses of adolescence, particularly when exercise is done in supportive social environments. Active adolescents, especially those who have encouragement from peers or mentors, are better equipped to withstand stress and achieve healthier emotional outcomes.

These findings convey a clear, actionable message to educators, healthcare providers, policymakers, and families: promoting physical activity is a practical means to improve adolescent mental health. Feasible strategies include providing daily opportunities for exercise in schools, integrating structured physical activity programs into youth mental health services, and supporting community initiatives that make sports and active recreation accessible to all young people. It is important to avoid a one-size-fits-all approach; the most effective programs will respect individual preferences, incorporate peer/mentor support, and ensure activities are enjoyable and mastery-oriented to build confidence and resilience. Although some specifics (such as the optimal activity type for certain outcomes or the best ways to engage the most inactive youth) are still being researched, the overarching evidence is clear and consistent: physical activity is a promising, low-cost, and empowering intervention that not only improves physical fitness but also significantly benefits mental well-being.

In conclusion, the relationship between physical activity, resilience, and mental health in adolescents is one of synergy and profound importance. An active body helps forge an active, adaptable mind. By embedding physical activity promotion within mental health frameworks (and conversely, integrating mental health considerations into physical activity programs), we can support adolescents in developing healthier minds and bodies together. This holistic approach offers hope for reversing negative trends in youth mental health and for nurturing a resilient next generation. As research continues to advance, it will further inform how we can best harness the power of movement to cultivate happier, more resilient young people across the globe. The imperative now is to translate this knowledge into real-world action—integrating physical activity into the very fabric of adolescent daily life and healthcare—so that every young person has the opportunity to build resilience and reach their full potential for mental well-being.

## References

[1] Patton GC Sawyer SM Santelli JS Ross DA Afifi R Allen NB. Our future: a lancet commission on adolescent health and wellbeing. Lancet. (2016) 387(10036):2423–78. 10.1016/s0140-6736(16)00579-1 27174304 PMC5832967

[2] Sawyer SM Afifi RA Bearinger LH Blakemore S-J Dick B Ezeh AC. Adolescence: a foundation for future health. Lancet. (2012) 379(9826):1630–40. 10.1016/S0140-6736(12)60072-5 22538178

[3] World Health Organization. Adolescent and young adult health (2024). Accessed online at: https://www.who.int/zh/news-room/fact-sheets/detail/adolescents-health-risks-and-solutions (Accessed November 26, 2024).

[4] Zhang G Feng WX Zhao LY Zhao XH Li TJ. The association between physical activity, self-efficacy, stress self-management and mental health among adolescents. Sci Rep. (2024) 14(1):5488. 10.1038/s41598-024-56149-4 38448518 PMC10917799

[5] Dumith SC Gigante DP Domingues MR Kohl HW III. Physical activity change during adolescence: a systematic review and a pooled analysis. Int J Epidemiol. (2011) 40(3):685–98. 10.1093/ije/dyq272 21245072

[6] Guthold R Stevens GA Riley LM Bull FC. Global trends in insufficient physical activity among adolescents: a pooled analysis of 298 population-based surveys with 1·6 million participants. Lancet Child Adolesc Health. (2020) 4(1):23–35. 10.1016/s2352-4642(19)30323-2 31761562 PMC6919336

[7] World Health Organization. Physical activity (2024). Accessed online at: https://www.who.int/news-room/fact-sheets/detail/physical-activity#:∼:text=encourages%20healthy%20growth%20and%20development,approximately%20US%24%2027 (Accessed June 26, 2024).

[8] Rodriguez-Ayllon M Cadenas-Sánchez C Estévez-López F Muñoz NE Mora-Gonzalez J Migueles JH. Role of physical activity and sedentary behavior in the mental health of preschoolers, children and adolescents: a systematic review and meta-analysis. Sports Med. (2019) 49(9):1383–410. 10.1007/s40279-019-01099-5 30993594

[9] Wanjau MN Möller H Haigh F Milat A Hayek R Lucas P. Physical activity and depression and anxiety disorders: a systematic review of reviews and assessment of causality. AJPM Focus. (2023) 2(2):100074. 10.1016/j.focus.2023.100074 37790636 PMC10546525

[10] White RL Babic MJ Parker PD Lubans DR Astell-Burt T Lonsdale C. Domain-specific physical activity and mental health: a meta-analysis. Am J Prev Med. (2017) 52(5):653–66. 10.1016/j.amepre.2016.12.008 28153647

[11] White RL Vella S Biddle S Sutcliffe J Guagliano JM Uddin R. Physical activity and mental health: a systematic review and best-evidence synthesis of mediation and moderation studies. Int J Behav Nutr Phys Act. (2024) 21(1):134. 10.1186/s12966-024-01676-6 39609855 PMC11603721

[12] Simpson A Teague S Kramer B Lin A Thornton AL Budden T. Physical activity interventions for the promotion of mental health outcomes in at-risk children and adolescents: a systematic review. Health Psychol Rev. (2024) 18(4):899–933. 10.1080/17437199.2024.2391787 39162060

[13] Deuster PA Silverman MN. Physical fitness: a pathway to health and resilience. US Army Med Dep J. (2013): 24–35. Available online at: https://europepmc.org/article/med/24146240 24146240

[14] Hoare E Skouteris H Fuller-Tyszkiewicz M Millar L Allender S. Associations between obesogenic risk factors and depression among adolescents: a systematic review. Obes Rev. (2014) 15(1):40–51. 10.1111/obr.12069 23980942

[15] Caspersen CJ Powell KE Christenson GM. Physical activity, exercise, and physical fitness: definitions and distinctions for health-related research. Public Health Rep. (1985) 100(2):126–31. Available online at: https://pmc.ncbi.nlm.nih.gov/articles/PMC1424733/3920711 PMC1424733

[16] Bouchard C Shephard RJ Stephens T. Physical Activity, Fitness, and Health: International Proceedings and Consensus Statement. Champaign, IL: Human Kinetics Publishers (1994). 10.1249/00005768-199401000-00024

[17] Corbin CB Pangrazi RP Franks BD. Definitions: Health, Fitness, and Physical Activity. President's Council on Physical Fitness and Sports Research Digest 3, no. 9. ERIC, ED470696. (2000). Available online at: https://eric.ed.gov/?id=ED470696 (Accessed April 28, 2026).

[18] Solmi M Basadonne I Bodini L Rosenbaum S Schuch FB Smith L. Exercise as a transdiagnostic intervention for improving mental health: an umbrella review. J Psychiatr Res. (2025) 184:91–101. 10.1016/j.jpsychires.2025.02.024 40043589

[19] Eisenmann JC. Aerobic fitness, fatness and the metabolic syndrome in children and adolescents. Acta Paediatr. (2007) 96(12):1723–9. 10.1111/j.1651-2227.2007.00534.x 17971189

[20] Windle G. What is resilience? A review and concept analysis. Rev Clin Gerontol. (2011) 21(2):152–69. 10.1017/S0959259810000420

[21] Lubans D Richards J Hillman C Faulkner G Beauchamp M Nilsson M. Physical activity for cognitive and mental health in youth: a systematic review of mechanisms. Pediatrics. (2016) 138(3):e20161642. 10.1542/peds.2016-1642 27542849

[22] Qiu WT Huang C Xiao HB Nie YY Ma WX Zhou FB. The correlation between physical activity and psychological resilience in young students: a systematic review and meta-analysis. Front Psychol. (2025) 16:1557347. 10.3389/fpsyg.2025.1557347 40365624 PMC12069377

[23] Older Adults Workgroup, Educational and Community-Based Programs Workgroup, Maternal, I., and Child Health Workgroup, Physical Activity Workgroup, & Arthritis Workgroup. Physical Activity Guidelines for Americans. 2nd Ed. Washington, DC: U.S. Department of Health and Human Services (2018). Available online at: https://health.gov/paguidelines/second-edition/pdf/Physical_Activity_Guidelines_2nd_edition.pdf (Accessed April 28, 2026).

[24] Valkenborghs SR Noetel M Hillman CH Nilsson M Smith JJ Ortega FB. The impact of physical activity on brain structure and function in youth: a systematic review. Pediatrics. (2019) 144(4):e20184032. 10.1542/peds.2018-4032 31554668

[25] Biddle SJ Asare M. Physical activity and mental health in children and adolescents: a review of reviews. Br J Sports Med. (2011) 45(11):886–95. 10.1136/bjsports-2011-090185 21807669

[26] Biddle SJH Ciaccioni S Thomas G Vergeer I. Physical activity and mental health in children and adolescents: an updated review of reviews and an analysis of causality. Psychol Sport Exerc. (2019) 42:146–55. 10.1016/j.psychsport.2018.08.011

[27] Greenwood BN Fleshner M. Exercise, stress resistance, and central serotonergic systems. Exerc Sport Sci Rev. (2011) 39(3):140–9. 10.1097/JES.0b013e31821f7e45 21508844 PMC4303035

[28] Brown HE Pearson N Braithwaite RE Brown WJ Biddle SJ. Physical activity interventions and depression in children and adolescents: a systematic review and meta-analysis. Sports Med. (2013) 43(3):195–206. 10.1007/s40279-012-0015-8 23329611

[29] Carter T Morres ID Meade O Callaghan P. The effect of exercise on depressive symptoms in adolescents: a systematic review and meta-analysis. J Am Acad Child Adolesc Psychiatry. (2016) 55(7):580–90. 10.1016/j.jaac.2016.04.016 27343885

[30] Cerrillo-Urbina AJ García-Hermoso A Sánchez-López M Pardo-Guijarro MJ Santos Gómez JL Martínez-Vizcaíno V. The effects of physical exercise in children with attention deficit hyperactivity disorder: a systematic review and meta-analysis of randomized control trials. Child Care Health Dev. (2015) 41(6):779–88. 10.1111/cch.12255 25988743

[31] Larun L Nordheim LV Ekeland E Hagen KB Heian F. Exercise in prevention and treatment of anxiety and depression among children and young people. Cochrane Database Syst Rev. (2006) 2006(3):CD004691. 10.1002/14651858.CD004691.pub2 16856055 PMC12742371

[32] Pascoe M Bailey AP Craike M Carter T Patten R Stepto N. Physical activity and exercise in youth mental health promotion: a scoping review. BMJ Open Sport & Exercise Medicine. (2020) 6(1):e000677. 10.1136/bmjsem-2019-000677 PMC7010991 32095272

[33] Smith JJ Eather N Morgan PJ Plotnikoff RC Faigenbaum AD Lubans DR. The health benefits of muscular fitness for children and adolescents: a systematic review and meta-analysis. Sports Med. (2014) 44(9):1209–23. 10.1007/s40279-014-0196-4 24788950

[34] Shomaker LB Tanofsky-Kraff M Zocca JM Field SE Drinkard B Yanovski JA. Depressive symptoms and cardiorespiratory fitness in obese adolescents. J Adolesc Health. (2012) 50(1):87–92. 10.1016/j.jadohealth.2011.05.015 22188839 PMC3245515

[35] Williams SE Carroll D Veldhuijzen van Zanten JJ Ginty AT. Anxiety symptom interpretation: a potential mechanism explaining the cardiorespiratory fitness-anxiety relationship. J Affect Disord. (2016) 193:151–6. 10.1016/j.jad.2015.12.051 26773908

[36] Kelly NR Mazzeo SE Evans RK Stern M Thacker LF Thornton LM. Physical activity, fitness and psychosocial functioning of obese adolescents. Ment Health Phys Act. (2011) 4(1):31–7. 10.1016/j.mhpa.2010.11.001

[37] Ortega FB Ruiz JR Castillo MJ Sjöström M. Physical fitness in childhood and adolescence: a powerful marker of health. Int J Obes. (2008) 32(1):1–11. 10.1038/sj.ijo.0803774 18043605

[38] LaVigne T Hoza B Smith AL Shoulberg EK Bukowski W. Associations between physical fitness and children's Psychological well-being. J Clin Sport Psychol. (2016) 10(1):32–47. 10.1123/jcsp.2014-0053

[39] Page RM Frey J Talbert R Falk C. Children’s feelings of loneliness and social dissatisfaction: relationship to measures of physical fitness and activity. J Teach Phys Educ. (1992) 11(3):211–9. 10.1123/jtpe.11.3.211

[40] Philippot A Meerschaut A Danneaux L Smal G Bleyenheuft Y de Volder AG. Impact of physical exercise on symptoms of depression and anxiety in pre-adolescents: a pilot randomized trial. Front Psychol. (2019) 10:1820. 10.3389/fpsyg.2019.01820 31440186 PMC6694801

[41] Evenson KR Catellier DJ Gill K Ondrak KS McMurray RG. Calibration of two objective measures of physical activity for children. J Sports Sci. (2008) 26(14):1557–65. 10.1080/02640410802334196 18949660

[42] Léger LA Lambert J. A maximal multistage 20-m shuttle run test to predict VO2 max. Eur J Appl Physiol. (1982) 49(1):1–12. 10.1007/bf00428958 7201922

[43] Léger LA Mercier D Gadoury C Lambert J. The multistage 20 metre shuttle run test for aerobic fitness. J Sports Sci. (1988) 6(2):93–101. 10.1080/02640418808729800 3184250

[44] Connor KM Davidson JR. Development of a new resilience scale: the connor-davidson resilience scale (CD-RISC). Depress Anxiety. (2003) 18(2):76–82. 10.1002/da.10113 12964174

[45] Gucciardi DF Hanton S Gordon S Mallett CJ Temby P. The concept of mental toughness: tests of dimensionality, nomological network, and traitness. J Pers. (2015) 83(1):26–44. 10.1111/jopy.12079 24428736

[46] Kmet LM Lee RC Cook LS. Standard Quality Assessment Criteria for Evaluating Primary Research Papers from a Variety of Fields. Edmonton, AB: Alberta Heritage Foundation for Medical Research (AHFMR) (2004). 10.7939/R37M04F16

[47] Campbell M McKenzie JE Sowden A Katikireddi SV Brennan SE Ellis S. Synthesis without meta-analysis (SWiM) in systematic reviews: reporting guideline. Br Med J. (2020) 368:l6890. 10.1136/bmj.l6890 31948937 PMC7190266

[48] Slavin RE. Best evidence synthesis: an intelligent alternative to meta-analysis. J Clin Epidemiol. (1995) 48(1):9–18. 10.1016/0895-4356(94)00097-a 7853053

[49] da Silva JM Castilho dos Santos G de Oliveira Barbosa R de Souza Silva TM Correa RC da Costa BGG. Effects of a school-based physical activity intervention on mental health indicators in a sample of Brazilian adolescents: a cluster randomized controlled trial. BMC Public Health. (2025) 25(1):539. 10.1186/s12889-025-21620-y 39930438 PMC11809091

[50] Shi YJ Zhao W Lipowski M. Effects of physical activity and resilience on emotional and behavioral problems in Chinese adolescent: a chained mediation model. Front Psychol. (2025) 16:1486949. 10.3389/fpsyg.2025.1486949 40693157 PMC12277388

[51] Duncan MJ Kuzik N Silva DAS Bélanger RE Carson V Chaput JP. Goldilocks days” for adolescent mental health: movement behaviour combinations for well-being, anxiety and depression by gender. Ment Health Phys Act. (2024) 26:100572. 10.1016/j.mhpa.2023.100572

[52] Silverman MN Deuster PA. Biological mechanisms underlying the role of physical fitness in health and resilience. Interface Focus. (2014) 4(5):20140040. 10.1098/rsfs.2014.0040 25285199 PMC4142018

[53] Lin H Zhu YY Liu QZ Li S. The mediating effect of resilience between physical activity and mental health: a meta-analytic structural equation modeling approach. Front Public Health. (2024) 12:1434624. 10.3389/fpubh.2024.1434624 39411497 PMC11473373

[54] Ekeland E Heian F Hagen KB Abbott J Nordheim L. Exercise to improve self-esteem in children and young people. Cochrane Database Syst Rev. (2004) 2004(1):CD003683. 10.1002/14651858.CD003683.pub2 14974029 PMC12935395

[55] Ellis WE Talebi S Dumas TM Forbes L. Adolescents’ physical activity and psychological adjustment across the first year of the COVID-19 pandemic. J Phys Act Health. (2022) 19(7):481–9. 10.1123/jpah.2022-0018 35894979

[56] Laurier C Pascuzzo K Jubinville V Lemieux A. Physical activity and its benefits on adolescents’ mental health through self-esteem. Front Child Adolesc Psychiatry. (2024) 3:1503920. 10.3389/frcha.2024.1503920 39816571 PMC11732102

[57] Noetel M Sanders T Gallardo-Gómez D Taylor P Del Pozo Cruz B van den Hoek D. Effect of exercise for depression: systematic review and network meta-analysis of randomised controlled trials. Br Med J. (2024) 384:e075847. 10.1136/bmj-2023-075847 38355154 PMC10870815

[58] Toseeb U Brage S Corder K Dunn VJ Jones PB Owens M. Exercise and depressive symptoms in adolescents: a longitudinal cohort study. JAMA Pediatr. (2014) 168(12):1093–100. 10.1001/jamapediatrics.2014.1794 25317674

[59] Stevens M Lieschke J Cruwys T Cárdenas D Platow MJ Reynolds KJ. Better together: how group-based physical activity protects against depression. Soc Sci Med. (2021) 286:114337. 10.1016/j.socscimed.2021.114337 34450391

[60] Carter T Guo BL Turner D Morres I Khalil E Brighton E. Preferred intensity exercise for adolescents receiving treatment for depression: a pragmatic randomised controlled trial. BMC Psychiatry. (2015) 15:247. 10.1186/s12888-015-0638-z 26467764 PMC4605143

[61] Li XN Wang J Yu HS Liu Y Xu XL Lin JB. How does physical activity improve adolescent resilience? Serial indirect effects via self-efficacy and basic psychological needs. PeerJ. (2024) 12:e17059. 10.7717/peerj.17059 38436018 PMC10909365

[62] Liu LQ Yisongwake A Hao Y Lyu Z Zhao ZH Wang ZC. The association between physical activity and positive affect in adolescents: the chain mediating role of psychological resilience and regulatory emotional self-efficacy. Psychol Health Med. (2024) 29(10):1807–19. 10.1080/13548506.2024.2411635 39377294

[63] Hoffmann MD Barnes JD Tremblay MS Guerrero MD. Associations between organized sport participation and mental health difficulties: data from over 11,000 US children and adolescents. PLoS One. (2022) 17(6):e0268583. 10.1371/journal.pone.0268583 35648742 PMC9159603

[64] Reardon CL Hitchcock M. Mental health in individual versus team sports. Int Rev Psychiatry. (2024) 36(3):284–95. 10.1080/09540261.2024.2349079 39255028

[65] Zhao JW Wang YF Yang C Guo PY. Commentary: the mediating effect of resilience between physical activity and mental health: a meta-analytic structural equation modeling approach. Front Public Health. (2024) 13:1599008. 10.3389/fpubh.2025.1599008 PMC12229840 40626157

[66] Li N Wang DG Zhao X Li Z Zhang L. The association between physical exercise behavior and psychological resilience of teenagers: an examination of the chain mediating effect. Sci Rep. (2024) 14(1):9372. 10.1038/s41598-024-60038-1 38654069 PMC11039466

[67] Ho FK Louie LH Chow CB Wong WH Ip P. Physical activity improves mental health through resilience in Hong Kong Chinese adolescents. BMC Pediatr. (2015) 15:48. 10.1186/s12887-015-0365-0 25898349 PMC4409716

[68] Liang X Qiu H Sit CHP. The mediating role of resilience in the association between MVPA and psychological ill-being in children with ADHD. Scand J Med Sci Sports. (2023) 33(4):485–94. 10.1111/sms.14282 36514869

[69] Liu C Yang YJ Wong SH Leung A Sit CH. The effects of physical activity on mental health in adolescents with attention-deficit hyperactivity disorder: a randomized controlled trial. Int J Behav Nutr Phys Act. (2025) 22(1):47. 10.1186/s12966-025-01745-4 40247314 PMC12007287

[70] Bang H Chang M Kim S. Team and individual sport participation, school belonging, and gender differences in adolescent depression. Child Youth Serv Rev. (2024) 159:107517. 10.1016/j.childyouth.2024.107517

[71] Kunitoki K Hughes D Elyounssi S Hopkinson CE Bazer OM Eryilmaz H. Youth team sports participation associates with reduced dimensional psychopathology through interaction with biological risk factors. Biol Psychiatry Glob Open Sci. (2023) 3(4):875–83. 10.1016/j.bpsgos.2023.02.001 37881582 PMC10593891

[72] Jiang WN Luo J Guan HN. Gender difference in the relationship of physical activity and subjective happiness among Chinese university students. Front Psychol. (2021) 12:800515. 10.3389/fpsyg.2021.800515 34950093 PMC8688753

[73] Xu P Chen JS Chang YL Wang XD Jiang XY Griffiths MD. Gender differences in the associations between physical activity, smartphone use, and weight stigma. Front Public Health. (2022) 10:862829. 10.3389/fpubh.2022.862829 35425758 PMC9001944

[74] Halliday AJ Kern ML Turnbull DA. Can physical activity help explain the gender gap in adolescent mental health? A cross-sectional exploration. Ment Health Phys Act. (2019) 16:8–18. 10.1016/j.mhpa.2019.02.003

[75] Ivanova MY Achenbach TM Turner L Almqvist F Begovac I Bilenberg N. Effects of individual differences, society, and culture on youth-rated problems and strengths in 38 societies. J Child Psychol Psychiatry. (2022) 63(11):1297–307. 10.1111/jcpp.13569 35167140

[76] Belcher BR Zink J Azad A Campbell CE Chakravartti SP Herting MM. The roles of physical activity, exercise, and fitness in promoting resilience during adolescence: effects on mental well-being and brain development. Biol Psychiatry: Cogn Neurosci Neuroimaging. (2021) 6(2):225–37. 10.1016/j.bpsc.2020.08.005 33067166 PMC7878276

[77] Teixeira PJ Carraça EV Markland D Silva MN Ryan RM. Exercise, physical activity, and self-determination theory: a systematic review. Int J Behav Nutr Phys Act. (2012) 9:78. 10.1186/1479-5868-9-78 22726453 PMC3441783

[78] Fortier MS Duda JL Guerin E Teixeira PJ. Promoting physical activity: development and testing of self-determination theory-based interventions. Int J Behav Nutr Phys Act. (2012) 9:20. 10.1186/1479-5868-9-20 22385751 PMC3353256

[79] Ntoumanis N Ng JYY Prestwich A Quested E Hancox JE Thøgersen-Ntoumani C. A meta-analysis of self-determination theory-informed intervention studies in the health domain: effects on motivation, health behavior, physical, and psychological health. Health Psychol Rev. (2021) 15(2):214–44. 10.1080/17437199.2020.1718529 31983293

[80] Manninen M Dishman R Hwang Y Magrum E Deng YY Yli-Piipari S. Self-determination theory based instructional interventions and motivational regulations in organized physical activity: a systematic review and multivariate meta-analysis. Psychol Sport Exerc. (2022) 62:102248. 10.1016/j.psychsport.2022.102248

[81] Li BW Ng K Tong XH Zhou X Ye JC Yu JJ. Physical activity and mental health in children and youth during COVID-19: a systematic review and meta-analysis. Child Adolesc Psychiatry Ment Health. (2023) 17(1):92. 10.1186/s13034-023-00629-4 37468975 PMC10357657

[82] Wright LJ Williams SE Veldhuijzen van Zanten J. Physical activity protects against the negative impact of coronavirus fear on adolescent mental health and well-being during the COVID-19 pandemic. Front Psychol. (2021) 12:580511. 10.3389/fpsyg.2021.580511 33776827 PMC7990778

[83] Hogan CL Catalino LI Mata J Fredrickson BL. Beyond emotional benefits: physical activity and sedentary behaviour affect psychosocial resources through emotions. Psychol Health. (2015) 30(3):354–69. 10.1080/08870446.2014.973410 25307453

[84] McCarthy N. WHO Warns 80% Of Adolescents Aren't Active Enough (2019). Accessed online at: https://www.statista.com/chart/20070/estimated-prevalence-of-insufficient-physical-activity/#:∼:text=Statista%20www,world%20are%20not%20moving%20enough (Accessed November 22, 2019).

[85] World Economic Forum. 80% of teenagers globally are too inactive—and it could shorten their lives (2019). Available online at: https://www.weforum.org/stories/2019/12/adolescents-youth-inactivity-exercise-health-obesity/#:∼:text=risk%20www,the%20first%20survey%20of (Accessed December 5, 2019).

[86] Bull FC Al-Ansari SS Biddle S Borodulin K Buman MP Cardon G. World health organization 2020 guidelines on physical activity and sedentary behaviour. Br J Sports Med. (2020) 54(24):1451–62. 10.1136/bjsports-2020-102955 33239350 PMC7719906

[87] Chaput JP Willumsen J Bull F Chou R Ekelund U Firth J. 2020 WHO guidelines on physical activity and sedentary behaviour for children and adolescents aged 5-1 7 years: summary of the evidence. Int J Behav Nutr Phys Act. (2020) 17(1):141. 10.1186/s12966-020-01037-z 33239009 PMC7691077

[88] Fuhrmann D Knoll LJ Blakemore SJ. Adolescence as a sensitive period of brain development. Trends Cogn Sci (Regul Ed). (2015) 19(10):558–66. 10.1016/j.tics.2015.07.008 26419496

[89] World Health Organization. Global Action Plan on Physical Activity 2018–2030: More Active People for a Healthier World. Geneva: World Health Organization (2018). Available online at: https://www.who.int/publications/i/item/9789241514187

[90] World Health Organization. Global Status Report on Physical Activity 2022. Geneva: World Health Organization (2022). Available online at: https://www.who.int/publications/i/item/9789240059153 (Accessed April 28, 2026).

[91] Biadgilign S Gebremichael B Abera A Moges T. Gender difference and correlates of physical activity among urban children and adolescents in Ethiopia: a cross-sectional study. Front Public Health. (2022) 10:731326. 10.3389/fpubh.2022.731326 35372220 PMC8965504

